# Protein's “Part-Time Job” Reveals New Facet of Signaling Pathway

**DOI:** 10.1371/journal.pbio.1001001

**Published:** 2010-11-23

**Authors:** Mason Inman

**Affiliations:** Freelance Science Writer, Karachi, Pakistan

**Figure:**
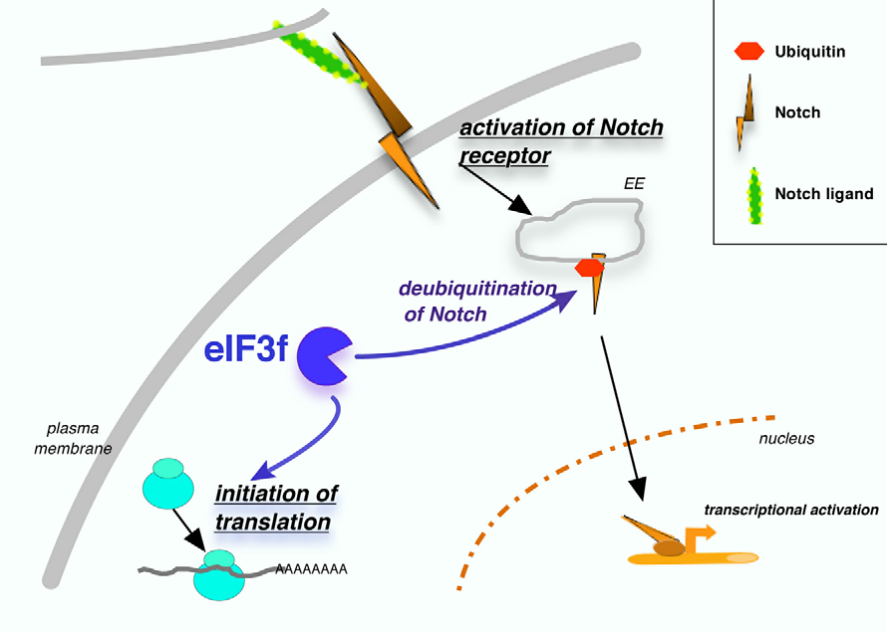
A dual role for eIF3f in the cell. eIF3f is necessary for initiation of translation and, in addition, acts positively on Notch signal transduction.

In a car, the key parts are usually specialized to do a specific job; the carburetor adjusts the mixture of air to gasoline that gets injected into the engine's cylinders, for example. But cells work differently, as a new study illustrates, with some proteins that have crucial jobs as “housekeeping proteins” also moonlighting with “part-time jobs” on the side.

As a new study in *PLoS Biology* shows, one such housekeeping protein turns out to have a side job in regulating the Notch signaling pathway, which all animals use for signaling between cells, and is crucial for normal development. This comes as a surprise, since that means the protein responsible has a side job that's arguably as crucial as its main job. And in uncovering this side job, the study also revealed a crucial step in the Notch pathway, which may allow researchers to understand better how that pathway works and how, when it goes haywire, it can cause disease.

Christel Brou of the Pasteur Institute in Paris and colleagues set out to find new enzymes that remove a widespread protein that attaches onto other proteins—it's so common, it's called ubiquitin. Adding a ubiquitin, or removing one, helps cells regulate many processes, so finding all the proteins that normally can add or remove ubiquitin, and how they work, is crucial for understanding regulation and signaling between cells.

It's been long known that Notch often carries one or more ubiquitin proteins on it. To dig deeper into how the removal of ubiquitin affects Notch signaling, Brou and colleagues screened all 91 enzymes that are known to remove ubitquitin, or are thought to have this ability based on their sequence of amino acids. The researchers used various combinations of short hairpin RNAs to successively knock down each one of the 91 known or suspected deubiquitinases, or DUBs (the enzymes that can remove ubiquitin). In this way, they were able to show that one of the suspected DUBs really does have this enzymatic skill—a protein known as eukaryotic translation initiation factor 3 subunit f (eIF3f).

The eIF3f protein was already known to have a crucial job as part of a larger set of several proteins that work together, known as the eIF3 complex. The eIF3 helps assemble a much larger complex of proteins essential for translation, the process of reading strands of RNA, and assembling proteins according to the RNA's code.

The new job for eIF3f that Brou and colleagues have uncovered is a crucial step in the Notch signaling pathway. Notch has a quirky way of passing signals between cells; it straddles the cell membrane, waiting for a trigger. When other proteins act on it in the right way, then Notch splits in two, releasing the part of Notch that dangles inside the cell. This part of Notch is then free to travel into the nucleus, where it acts as a transcription activator, which helps genes get expressed.

This whole process allows a signal from outside the cell to be passed into the nucleus, changing the cell's gene expression—a crucial part of allowing neighboring cells to coordinate their development. Problems in the Notch pathway can cause cancer, and has also been implicated in liver disease and a certain type of mental retardation.

By studying when and where eIF3f does its “side job,” Brou and colleagues found that it plays a crucial role in Notch signaling. With the help of another protein called Deltex1, which acts as a matchmaker, eIF3f associates with Notch when it is in the cell membrane, carrying a single ubiquitin protein. Then eIF3f removes the ubiquitin, preparing Notch for the next step, when a complex called gamma secretase cleaves Notch in two, releasing the inner part to travel into the cell nucleus. If eIF3f is mutated or otherwise knocked out of normal activity, the new study found, then this disrupts normal Notch signaling, showing eIF3f is essential for normal signaling.

The eIF3f protein isn't alone in having a side job. An earlier study showed that another transcription initiation factor, eIF4A, also seems to have side job as an enzyme that can add ubiquitin onto proteins. Likewise, other studies have shown that parts of the ribosome—the protein complex that builds new proteins—also have side jobs. So the new study suggests that while housekeeping proteins are already busy, having other roles to play may be more common than thought before. And if the new study is anything to go by, searching out these proteins' side jobs may reveal new facets of the workings of crucial systems in the cell.

**Moretti J, Chastagner P, Gastaldello S, Heuss SF, Dirac AM, et al. (2010) The Translation Initiation Factor 3f (eIF3f) Exhibits a Deubiquitinase Activity Regulating Notch Activation. doi:10.1371/journal.pbio.1000545**

